# Supplementary material for: Hyperexpression of CDRs and HWP1 genes negatively impacts on Candida albicans virulence
Source: PLoS One. 2021 Jun 1;16(6):e0252555. doi: 10.1371/journal.pone.0252555 (PMC8168907; doi:10.1371/journal.pone.0252555)
Supplement: Attachment — Submitted filename: Response to reveiwers.docx [file pone.0252555.s001.docx]

Dear Editor,

we are pleased to send you a fully revised version of the article: “Hyperexpression of CDRs and HWP1 genes negatively impacts on *Candida albicans* virulence”, modified to take into account all suggestions and critics of the reviewers and editor request regarding funding information.

We believe that now data are reported in a much more clear and exhaustive way. We hope you can now find our study suitable for publication in PlosOne.

Best regards

Letizia Angiolella

Following the detailed answers to the referees points:

**General points.**

i) English has been revised as well as mis-typings all over the text.

**Referee 1**

**Point 1 Mis-typing:** See general point.

**Point 2 Temperature condition:** we apologize for the mistake**.** Temperature conditions of the solid media for adapting and the liquid media for MIC were both set at 28° C. Corresponding sentence in Material and Methods, at line 109, has been amended, accordingly.

**Point 3 Echinocandin status:** the CI1 and CI2 isolates were assayed for caspofungin and MIC value was 8 μg/mL for both isolates, overlapping the results obtained for micafungin.

**Point 4 CDR1 upregulation in RFK:** we agree that the increase of CDR1 and CDR2 in micafungin resistant strain is not explained with the efflux mechanism usually involved in fluconazole resistant. However, an increase in CDR1 and 2 has been reported as associated with a caspofungin resistance in *Candida albicans* clinical isolates (Schuetzer-Muehlbauer M, Willinger B, Krapf G, Enzinger S, Presterl E, Kuchler K. The *Candida albicans* Cdr2p ATP-binding cassette (ABC) transporter confers resistance to caspofungin. Mol Microbiol. 2003 Apr;48(1):225-35. doi: 10.1046/j.1365-2958.2003.03430.x). To confirm CDRs role, authors expressed these genes in *Saccharomyces cerevisiae* and observed an increased MIC for caspofungin. It is worth notice that the authors stated that resistance is probably not due to the direct effect of these proteins as efflux pumps but more reasonably: “as a consequence of distinct mechanisms that may operate simultaneously”. A similar scenario may be tentatively proposed for our micafungin resistant strain.

**Point 5 CDR3 and CDR4:** we missed the analyses for these two drug transporters because, to our knowledge, they seem not to have a critical role in resistance to fluconazole. CDR3 was found highly expressed in WO-1 opaque cells and its overexpression was not linked to an increase in fluconazole resistance. This data suggested a role for this protein in the white-opaque switch rather than in drug efflux. (Balan I, Alarco AM, Raymond M. The *Candida albicans* CDR3 gene codes for an opaque-phase ABC transporter. J Bacteriol. 1997 Dec;179(23):7210-8. doi: 10.1128/jb.179.23.7210-7218.1997.). A similar consideration may be proposed for CDR4. CDR4 mutant revealed not hypersusceptible to fluconazole and furthermore Candida isolates from AIDS patients treated with fluconazole did not reveal any involvement of CDR4 in resistance (Franz R, Michel S, Morschhäuser J. A fourth gene from the *Candida albicans* CDR family of ABC transporters. Gene. 1998 Oct 5;220(1-2):91-8. doi: 10.1016/s0378-1119(98)00412-0.)

**Point 6 RT-PCR internal control:** We tried to assess the expression rate using 18S rRNA as housekeeping gene for CDR1 and CDR2 (unfortunately, we had not a suitable amount left for screening the other genes). Analyses showed a similar pattern obtained with the 18S rRNA to that reported in the paper obtained with actin gene. Furthermore, these results agree with those reported in a previous paper where TEF3 gene was used as housekeeping (Angiolella L, Stringaro AR, De Bernardis F, Posteraro B, Bonito M, Toccacieli L, Torosantucci A, Colone M, Sanguinetti M, Cassone A, Palamara AT. Increase of virulence and its phenotypic traits in drug-resistant strains of *Candida albicans*. Antimicrob Agents Chemother. 2008 Mar;52(3):927-36. doi: 10.1128/AAC.01223-07). Data are reported in the figures below. (Please note that only two replicates for CDR2 were performed due to the small availability of this sample and thus statistical analysis was missed).

**B**

**A**

**A**

**D**

**C**

**B**

**Expression levels of *C. albicans*  CDR1 and CDR2 genes determined by quantitative Real-Time PCR.**

The expression levels of the CDR1 and CDR2 with Actin as housekeeping (A and C) and CDR1 and CDR2 with 18S rRNA as houskeeping (B and D) in the wild-type (CO23), resistant (CO23RFK, CO23RFLC and CO23RR) and clinical isolated (CI1 and CI2) strains, represented as n-fold increase or decrease relative to the level of the control strain (CO23). Data for CDR1s and CDR2 with Actin housekeeping and for CDR1 with 18S rRNA are shown as mean±SEM from three independent experiments performed in triplicate. **p*< 0.05, ***p*< 0.01, ****p*< 0.001, *****p*<0.0001 *vs* CO23_S_ and ^#^*p*< 0.05, ^##^*p*< 0.01, ^###^*p*< 0.001, ^####^*p*< 0.0001 *vs* CO23_RR_.

**Point 7 Calculation of relative expression profiles:** A comparative threshold cycle (CT) method was used to analyze the RT-PCR data. Sensitive *Candida albicans* sample (CO23s) was used as a control and target gene Ct values were normalized against actin. Data were analyzed by using the 2^-ΔΔCT^ method and expressed as fold change with respect to control (Livak KJ, Schmittgen TD. Analysis of relative gene expression data using real-time quantitative PCR and the 2(-Delta Delta C(T)) Method. Methods. 2001 Dec;25(4):402-8. doi: 10.1006/meth.2001.1262.). This sentence has been added in Material and Method in section: Extraction of RNA and RT-PCR on lines 131-134

**Point 8 MSF-QDR:** a sentence suggesting an involvement of QDR family in the remarkable hypha development observed in CI1 has been added in the Discussion, lines 311-314 along with the reference (Shah AH, Singh A, Dhamgaye S, Chauhan N, Vandeputte P, Suneetha KJ, Kaur R, Mukherjee PK, Chandra J, Ghannoum MA, Sanglard D, Goswami SK, Prasad R. Novel role of a family of major facilitator transporters in biofilm development and virulence of *Candida albicans*. Biochem J. 2014 Jun 1;460(2):223-35. doi: 10.1042/BJ20140010.)

).

**Point 9 Mutations:** genetic analysis of fluconazole resistant strains was missing. We analysed RT-PCR expression of ERG11 in sensitive, flucanozole and micafungin resistant strains and we found no difference in gene expression between these strains (Angiolella L, Stringaro AR, De Bernardis F, Posteraro B, Bonito M, Toccacieli L, Torosantucci A, Colone M, Sanguinetti M, Cassone A, Palamara AT. Increase of virulence and its phenotypic traits in drug-resistant strains of *Candida albicans*. Antimicrob Agents Chemother. 2008 Mar;52(3):927-36. doi: 10.1128/AAC.01223-07.). In the same paper, we reported a S645Y mutation on FSK1 gene in micafungin resistant strain.

**Referee 2**

**Point 1 Data do not support conclusion** Overexpression of CDRs and MDR is a usual event occurring in fluconazole resistant strains. The increased expression of this efflux pumps is usually correlated with an increased virulence of the fungus. In this paper we found an evidence, the hyperexpression of these genes and HWP1, that opposes to this common view. For this reason, we believe that this report may be a worthwhile information in this research field.

**Point 2 Discussion is speculative** Unfortunately, data available in literature on regulatory pathways responsible for altered gene expression and reported in the Discussion, do not allow to find a common link in the regulation of the hyperexpression of the three analysed genes. Therefore we refrained from fully correlate reduction of Candida lethality to the altered gene expression rather suggesting that it may be just an epiphenomenon of a vaster rearrangement occurring in these strains.

**Point 3 Virulence genes** We agree that the panel of virulence genes could be increased. However, focusing on gene expression, CDRs and MDR are frequently overexpressed in fluconazole resistant strains along with ALS1 and HWP1 as far as filamentation and adhesiveness is concerned.

**Point 4 Number of clinical strains** We believe that the clinical isolate with the hyperexpression of CDR1, CDR2 and HWP1, found by serendipity, represents a very rare event and then it is unlikely to fish again a resistant clinical strains with a similar phenotype even increasing the number of clinical strains analysed.

**Point 5-6: Abbreviations -Tables and figures** We agree that it is difficult to memorize all strain abbreviations and therefore we decided to insert a new Table (Table 2) in the Result section, reporting information and abbreviation for each strain.

**Point 7-8 English**: see general points.

**Funding information:** we delete in the Acknowledgements section thanks to “Cooperativa Comunale Logistica Industriale Integrata” without sending the founding statement and therefore competing interest statement remain the same as previously.

Founders’ roles have been updated.
